# The democratic problem in the knowledge society of today: Rare Disorders Denmark’s documentation strategy

**DOI:** 10.1186/1750-1172-7-S2-A35

**Published:** 2012-11-22

**Authors:** Ane Lind, Lene Jensen, Birthe B Holm

**Affiliations:** 1 Rare Disorders Denmark, Copenhagen, DK-1220, Denmark

## Knowledge society

In the knowledge society of today, scientific knowledge is considered as objective and based on evidence, while experience-based knowledge which relates to patients and their organisations is considered as subjective, based on emotions and self-interest. Thus, experience-based knowledge does not fit the norm of scientific knowledge and informed decision making.

This may be even more dominant within healthcare. It reflects a democratic problem as it can be regarded as a structural censorship that some kinds of knowledge is reflected to be truer than others.

Knowledge production by small NGO´s is disfavoured, since they do not have resources to produce evidence-based knowledge. Furthermore, the conditions to create evidence in small populations are difficult.

## The documentation strategy

Rare Disorders Denmark (RDD) is a national alliance for patient organisations for people with rare disorders. RDD believes that patients hold unique experience-based knowledge - important knowledge that cannot be obtained elsewhere. But to have experience-based knowledge recognised in society, we see a substantial need to document our knowledge in a scientific way with methods dominating the field of interest.

Systematic documentation has following advantages:

- Documenting the effects of our activities and policies

- Recognition by professionals and decision makers

- An entry to make alliances with professionals

- Credibility

By documenting experience-based knowledge and activities, we believe professionals and decision makers are more likely to recognise this knowledge. In the documentation process we often invite patient representatives and professionals to take part in an advisory board. This is an entry to make alliances with professionals and qualify our results. Furthermore the advisory board has a mediating function as professionals from related areas and patients meet in a relaxed environment, with the opportunity to exchange knowledge and viewpoints. In addition, results are easily disseminated to members of the advisory board and having researchers, professors, specialists, etc. in the board contribute to the credibility of the used methods and results.

By documenting our knowledge RDD takes form of a serious, professional operator of knowledge, recognised by professionals, even though RDD’s identity is grounded in patients’ experience-based knowledge.

A recent example of RDD’s documentation strategy is the on-going project, Rare Family Days, which is described in another abstract in the present journal edition.

